# Livedoid Vasculopathy: diagnosis and treatment in pregnant women

**DOI:** 10.1590/1677-5449.190093

**Published:** 2020-06-01

**Authors:** Alexandre Sacchetti Bezerra, Accácio de Almeida Abussamra Junqueira de Andrade, Afonso César Polimanti, Rafael Vilhena de Carvalho Fürst, Paulo Ricardo Criado, João Antônio Corrêa

**Affiliations:** 1 Faculdade de Medicina do ABC, Disciplina de Angiologia e Cirurgia Vascular, São Paulo, SP, Brasil.; 2 Universidade de São Paulo (USP), Disciplina de Dermatologia, São Paulo, SP, Brasil.

**Keywords:** anticoagulants, atrophy, vasculitis, anticoagulantes, atrofia, vasculite

## Abstract

Livedoid Vasculopathy is a disease characterized by occlusion of the capillaries of the dermis, without inflammatory signs. It begins with purpuric papules or macules that develop into painful ulcers, mainly involving the ankles and feet. In this case report, we describe diagnosis and treatment in a young pregnant patient, with excellent clinical response.

## Resumo

A vasculopatia livedoide é uma doença caracterizada pela oclusão dos capilares da derme, sem sinais inflamatórios. Tem início com pápulas ou máculas purpúricas que evoluem para úlceras dolorosas, com predominância na topografia de tornozelos e pés. Neste relato de caso, descrevemos o diagnóstico e a terapêutica em uma paciente jovem gestante, com excelente evolução clínica.

## INTRODUCTION

Livedoid Vasculopathy (LV) is a cutaneous disease involving occlusion of dermal vessels.^1^^,^^2^

In the past, this condition was known as Livedoid Vasculitis. However, in 2003, Zanini et al.^1^ documented the absence of characteristics of Primary Vasculitides.^1^^,^^3^

In contrast with primary vasculitis, no consumptive syndrome or vascular lesions with primary immunological modulation are observed in this disease.^3^^,^^4^

If immunological changes are present in vasculopathies, they are secondary. In the majority of cases, histopathological studies do not detect an inflammatory infiltrate associated with karyorrhexis.^3^^,^^5^

Even without defined pathophysiology, LV presents painful macules or papules in the lower limbs, predominantly in the feet and ankles. The pain is relevant, because it marks progression or remission of the disease during treatment.

Painful lesions develop into ulcers associated with scarring known as Milian’s Blanche Atrophy and which are usually related to livedo racemosa. There is also disease progression in the summer, termed by some as “[…] vasculitis with ulceration in the summer”.^2^^,^^5^

This rare vasculopathy affects 1 to 100,000 inhabitants per year and is three times more prevalent among women aged between 15 and 50 years.^2^^,^^3^^,^^5^

There are no reports in the literature of patients who became pregnant while being treated for LV.

The present study describes a clinical case of LV in a young patient, who became pregnant after diagnosis and initiation of therapy.

## CASE REPORT

The patient was a 34-year-old married woman.

She sought care at a public hospital for painful ulcers associated with macules involving the left ankle, with onset 3 months previously. She rated her pain as of high intensity, worsening with low temperatures.

The patient had not achieved improvement of the painful condition through use of nonsteroidal anti-inflammatory drugs and analgesics, administered without medical guidance.

The patient stated she had no comorbidities or other diseases and reported no similar cases in the family.

**Figure 1:**
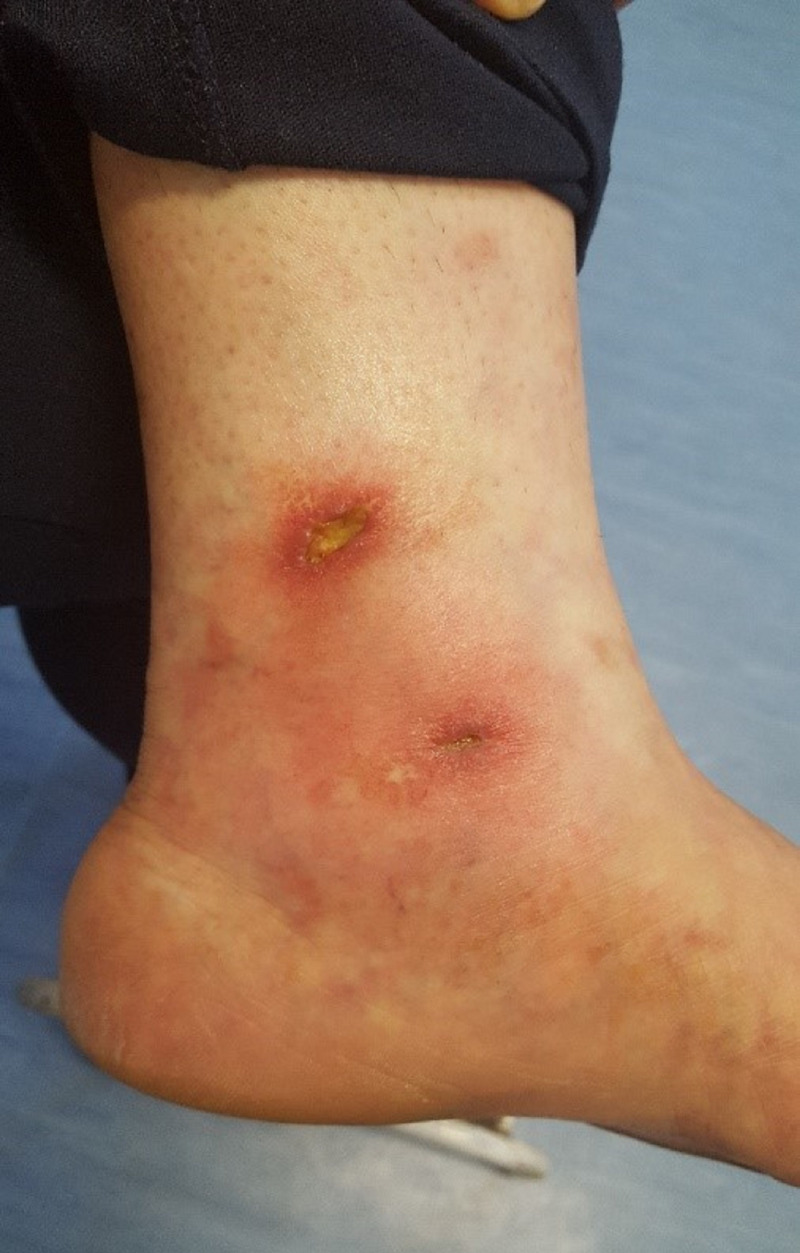
Livedo racemosa.

The physical examination, documented in Figure 1, found livedo racemosa involving the lower left limb, and ulcers with hyperemic borders with fibrin, hyperemia and dermatosclerosis. Distal pulses were present, symmetrical, and normal.

A biopsy of the ulcerated lesions performed on October 6, 2017, resulted in the following diagnosis:

- “Fragment of skin exhibiting epidermis with preserved maturation. In the underlying dermis, there is superficial perivascular lymphomononuclear infiltrate, sometimes permeating the vessel wall. Thickening of the vessel walls with thrombi. Absence of elements of malignancy. The histological features are compatible with the clinical hypothesis of Livedoid Vasculitis”.

Screening for inherited and acquired thrombophilia was performed, testing for Factor V Leiden, Prothrombin G20210A, Hiperhomocysteinemia, Protein S and C deficiencies, anticardiolipin antibodies, anti-beta2-glycoprotein I antibodies, and lupus anticoagulant and assaying prothrombin time and activated partial thromboplastin time.

All results of laboratory tests for possible thrombophilias were negative.

Diagnoses of other acquired thrombophilias, such as cancer, were ruled out by patient history, physical examination, and imaging studies.

For personal reasons, the patient did not attend the follow-up appointment, returning on December 15, 2017, untreated, and reporting worsening of pain. Treatment was started with 100 mg/day acetylsalicylic acid, low weight heparin, warfarin, and 200 mg/day cilostazol.

**Figure 2:**
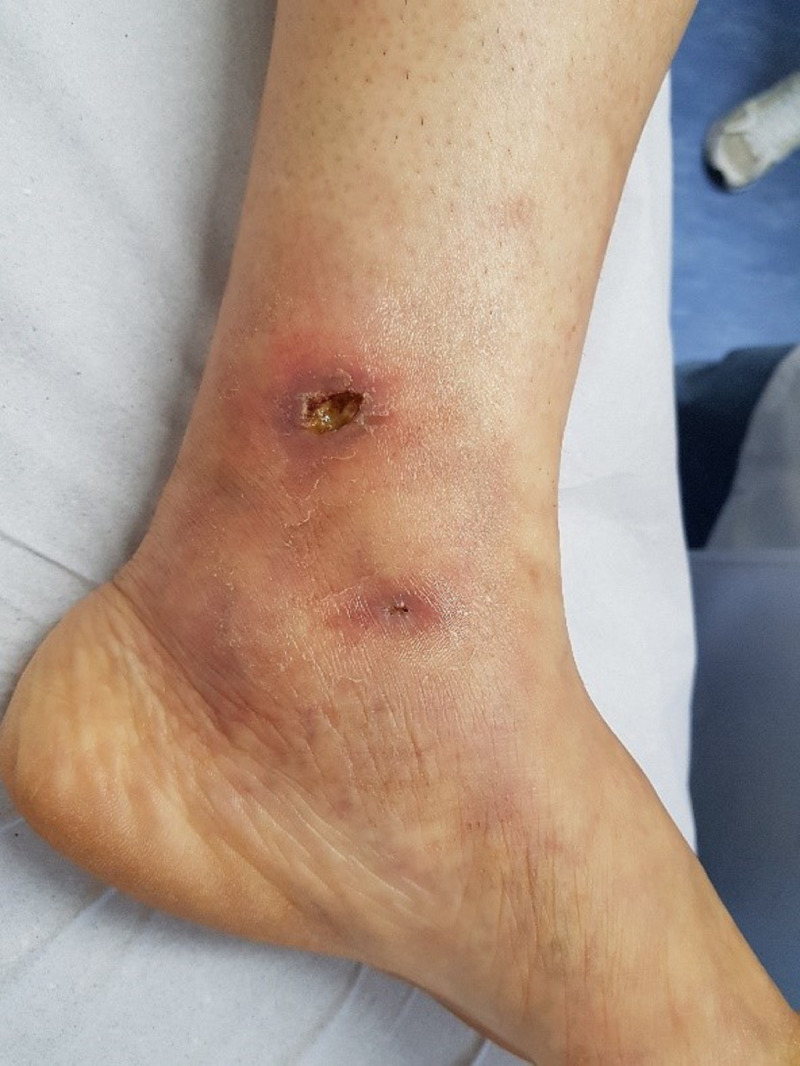
Regression of Milian’s Atrophy.

On December 29, 2017, fourteen days after starting the treatment described above, the patient presented total resolution of pain, regression of hyperemia of ulcers, and signs of healing. There was regression of the livedo racemosa that extended all over the foot. Figure 2 shows these changes, in addition to a reduction in Milian’s atrophy. Within this scenario, the patient reported that it was her intention to become pregnant, which was contraindicated by the medical staff.

**Figure 3:**
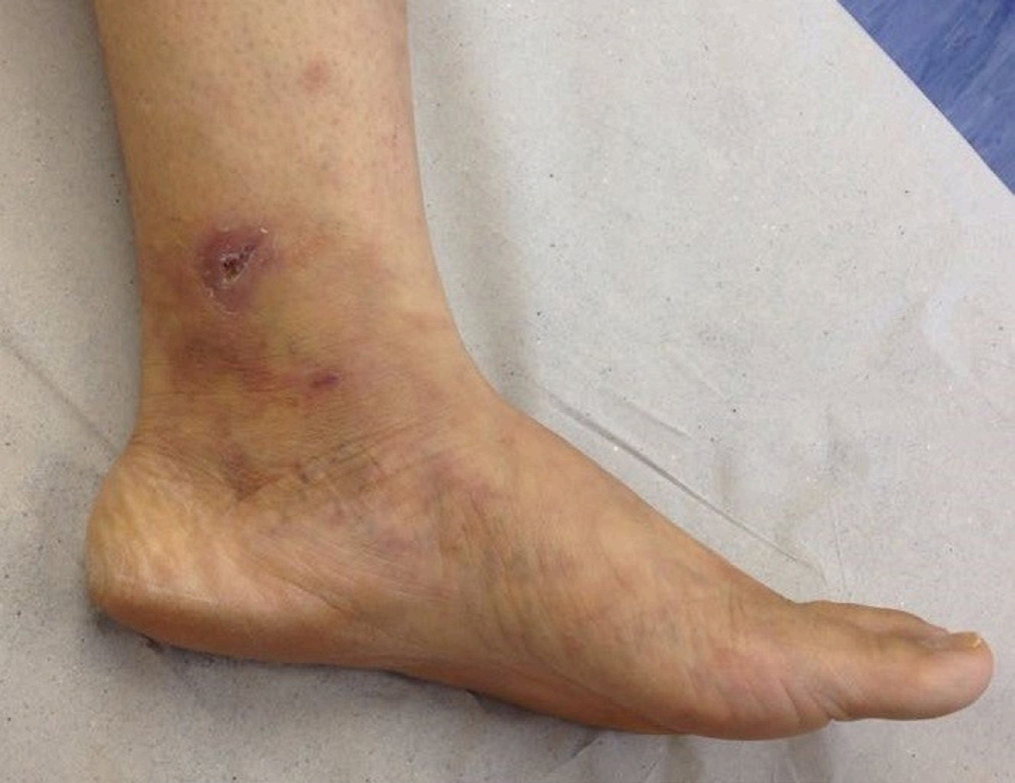
Complete regression.

Figure 3 shows the patient’s condition at a physical examination conducted on February 9, 2018, with healing of the ulcers, complete regression of the livedo racemosa, and no complaint of pain.

Against medical advice, the patient reported maintaining sexual activity without using a contraceptive method. Also on February 9, 2018, a diagnosis of pregnancy was confirmed after requesting a human chorionic gonadotropin test.

The 5mg/day warfarin was withdrawn immediately and low weight heparin was initiated at 120 mg/day.

**Figure 4:**
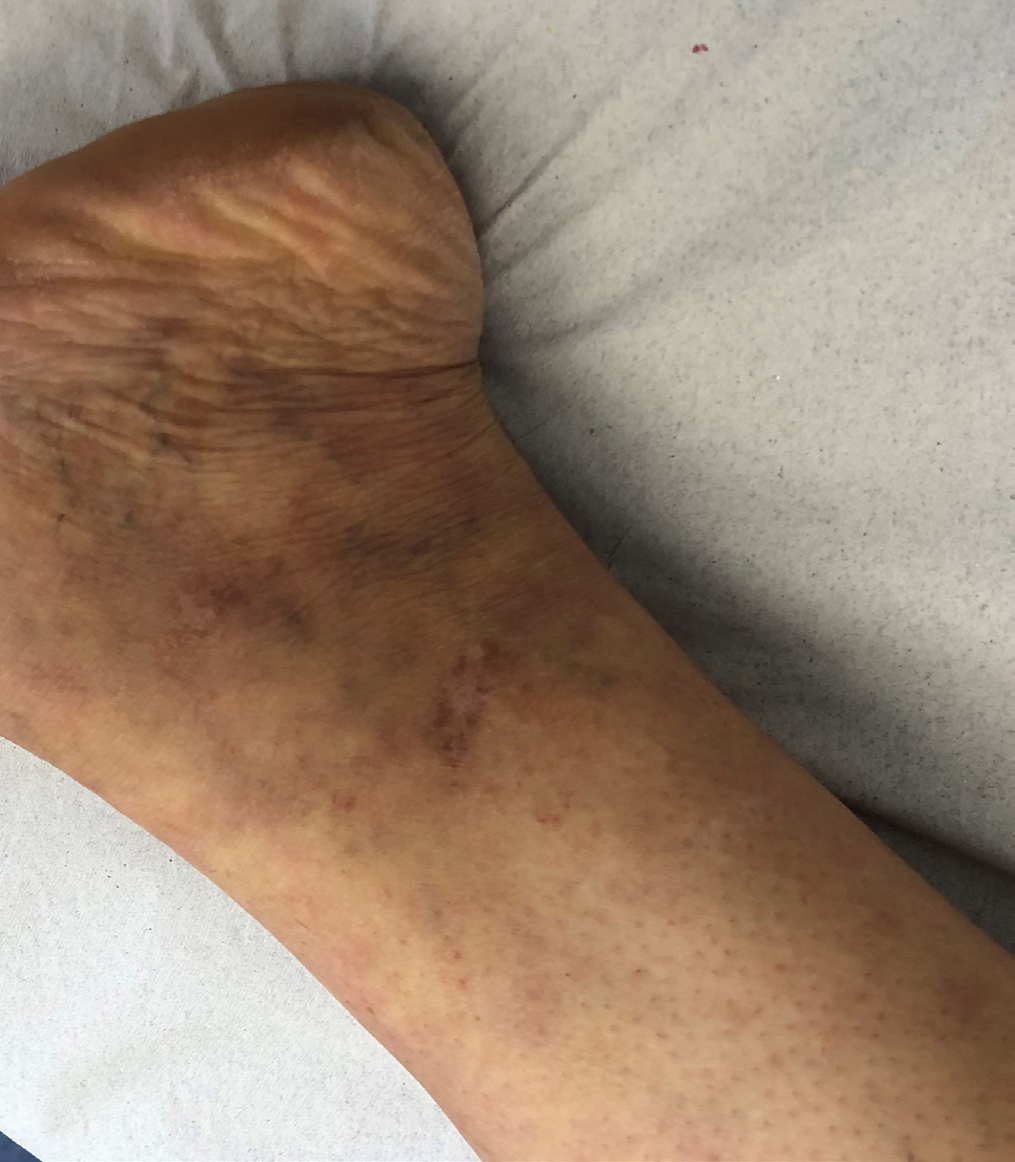
March 15, 2019.

During the pregnancy, there was no edema in the left lower limb, venous dilation, varicose veins, dermatosclerosis, phlebitis, deep venous thrombosis, or other signs (Figure 4).

On October 5, 2018, the patient had morphological USG findings compatible with 38 weeks of pregnancy. The lower left limb physical examination findings were unchanged.

The second gestation was delivered on October 13, 2018, with no complications.

Since the clinical condition had been resolved, daily prophylactic anticoagulation was administered with 40 mg/day of low weight heparin for just 10 days after delivery.

Analysis of ultrasound examinations and clinical data showed that the patient had not been pregnant at the start of treatment.

A physical examination performed on March 15, 2019, found no evidence of acute signs compatible with recurrence of LV.

## DISCUSSION

LV is a disease with unknown etiology.^2^^,^^3^

In addition to vascular involvement, patients with LV also have nervous system involvement, possibly caused by deposits of fibrin and thrombin in the vasa nervorum.^6^

The disease may be classified as idiopathic or secondary.^3^^,^^7^

Secondary disease can be induced by both coagulopathies and autoimmune connective tissue disorders. In order to do so, one should investigate the patient in question as to the possible causes, and Idiopathic Livedoid Vasculopathy is diagnosed by exclusion.^5^^,^^7^

A number of possible differential diagnoses must be taken into account. Of these, chronic venous insufficiency, obstructive peripheral arterial disease, microangiopathies, and vasculitides have been highlighetd.^1^^,^^3^^,^^4^^,^^8^

Chronic venous insufficiency induces painful ulcers, but they are usually exudative and with varicose components, and can be confirmed by simple physical examination.

Obstructive peripheral arterial disease also causes ulcers that heal with difficulty and are painful. In this case, simple diagnostic tests, such as ankle-brachial index and palpation of pulses are sufficient.

Vasculitides, such as Polyarteritis Nodosa, are a challenge for differential diagnosis and it is imperative to conduct a biopsy.^3^^,^^8^

After LV has been diagnosed, it is important to investigate its etiology, with work up tests to investigate thrombophilia, autoimmune diseases, cancer, peripheral vascular diseases, etc.^3^^,^^5^

Broad spectrum treatment aims to resolve the symptoms, the causes, and especially the thrombotic process. Treatments described in the literature consist of antiplatelet drugs, anticoagulants, fibrinolytics, hyperbaric chamber, vasodilators, and immunosuppressants.^3^^,^^5^^,^^9^

Warfarin use is forbidden in pregnant women, except patients with a metallic valve, due to its adverse effects.^10^

Vitamin K Inhibitors are linked with spontaneous abortion, and multiple teratogenic complications in pregnancy, such as ventral and dorsal midline dysplasias, limb hypoplasia, stippled epiphyses, and nasal hypoplasia.^10^^,^^11^

Oral direct thrombin inhibitors (dabigatran) and factor Xa inhibitors (rivaroxaban, apixaban, and edoxaban) cross the placenta. Their use during pregnancy is reported to increase gestational risk in animal studies, and safety is unclear from pregnant human trials.^11^^-^13

There are no guidelines for medical treatment of LV in either pregnant or non-pregnant patients.^3^^,^^5^

Because the patient in question had not previously undergone treatment, we chose not to use corticosteroids, fibrinolytics, or immunosuppressants.

Notwithstanding regression of livedo racemosa and complete healing of ulcers, pain measurement remains the best parameter of treatment efficacy. In the case described here, the patient reported absence of pain after just fourteen days of treatment.^2^^,^^3^^,^^6^

There are currently reports of use of low-dose fibrinolytic for patients refractory to initial or recurrent therapy. Deng et al.^14^ used rt-PA (alterase) at a dose of 10 mg/day for 14 days, combined with 10,000 IU/day of subcutaneous heparin and 81 mg/day of acetylsalicylic acid.

However, the risk of hemorrhagic accidents and the need for hospitalization restricts use of fibrinolytics to cases of failure of initial therapy or relapse. Immunosuppressants are usually used in refractory or recurrent cases.^9^^,^^14^

Despite the countless factors involved in this case, we achieved therapeutic success.

To date, we have not been able to find in the literature another report of pregnancy occurring during treatment for Livedoid Vasculopathy.
